# A Survey of Safety Recommendations for All-Terrain Vehicle Dealers and Track Owners in Kansas

**Published:** 2017-11-30

**Authors:** Morgan J. Martin, Rychael Morton, Shawn Rau, Sue Nyberg, Gina M. Berg

**Affiliations:** 1 Wichita State University, Department of Physician Assistant, Wichita, KS; 2 University of Kansas School of Medicine-Wichita, Department of Family and Community Medicine, Wichita, KS; 3 Wesley Medical Center, Wichita, KS

**Keywords:** all terrain vehicles, safety, prevention

## Abstract

**Introduction:**

All-terrain vehicles (ATVs) are associated with injury, mortality, and healthcare costs. ATV related injuries are less severe when consistent safety practices are followed, however, ATV safety regulations are varied among states. This study sought to survey Kansas ATV dealers and track owners to determine safety promotion practices.

**Methods:**

A cross-sectional telephone survey was conducted of Kansas ATV dealers and tracks. Survey questions included promotion and sale of safety equipment, provision of ATV safety information, and respondent characteristics.

**Results:**

Of those contacted, 32% of dealers and 31% of tracks responded to the survey. Most ATV dealers sell safety gear (70% – 100%) and all recommend safety gear to buyers and riders. All ATV tracks reported requiring helmets (100%) but were varied regarding other forms of safety gear. The majority of ATV dealers (77%) recommended safety courses, but only 31% of dealers and 40% of tracks offered courses. Eighty percent of ATV tracks and 52% of dealers felt they had a professional responsibility to educate riders/owners on safety.

**Conclusion:**

Safety promotion by ATV dealers in Kansas consistently was recommended, but often limited to the sales of safety gear (helmets and gloves) or the provision of manufacturer provided safety materials. Further, ATV dealers reported rarely offering skills tests or safety courses to buyers. In Kansas, safety promotion at the point of sale or track level could be improved to increase public awareness of ATV safety practices.

## Introduction

All-terrain vehicles (ATVs) are defined as any motorized vehicle with three or four low-pressure tires, a straddle seat, and a handle bar.^1^ Models can vary in size and power with engine capabilities upwards of 400 cubic centimeters (cc), which may achieve speeds up to 70 miles per hour (mph). ATVs are used both commercially (farming and ranching) and recreationally. Commercially, ATVs are used more often by youths (younger than 16) than tractors.^2^ However, recreational use has been related to more injury^2^ and noted to be more dangerous than motocross^3^, dirt bikes, and snowmobiles.^4^

Use of ATVs has been associated with significant injury, mortality, and healthcare cost.^5^ Reported ATV related injuries include: bone fractures at or below the cervical spine, specifically femur and tibia^6^, upper extremities, thoracic, peripheral nerve, and soft tissue injuries^4^ and traumatic brain injuries.^4^,^7^ A recent national review of ATV fatalities reported a rate of .32 per 100,000^8^; while Garay and colleagues^6^ observed a 1.5% mortality rate among all pediatric ATV injuries in Pennsylvania. Hospital costs associated with ATV related injuries were reported upwards of $300,000, with a mean cost of approximately $33,000.^5^

Many of these injuries could be prevented by using safety equipment such as helmets, gloves, boots, goggles, chest protectors, knee pads, and elbow pads.^9^ Fatalities, injury severity scores and incidence of traumatic brain injury incidence decreased when riders wore helmets.^7^,^10^–^12^ Keenan and Bratton^13^ compared injuries between Pennsylvania (helmet law and road restrictions) to North Carolina (no restrictions) and observed that restrictions were associated with decreased ATV related injuries. As of 2014, the National Conference of State Legislatures^14^ reported the following state laws regarding ATV use: 34 states required helmet and/or eye protection, 34 states mandated a minimum age for ATV operation ranging from 6–18 years old, 23 states required an education course for ATV riders. Kansas, however, had none of these laws in place regarding ATV use. The three Kansas state laws regarding ATV use include: ATVs must be titled, ATVs may not be operated on an interstate, federal, or state highway, and ATVs must be equipped with headlights and taillights.^15^ Helmet use for three-wheel ATVs is in accordance with Kansas motorcycle laws in that riders under age 18 must wear a helmet.^16^

ATV safety may be dependent on the safety campaigns and promotion of public awareness through influential change agents associated with ATV use. Jennissen and colleagues^17^ evaluated a safety awareness initiative targeting agribusinesses and found that most did or would have posted the safety material (if received). Another target for safety awareness could be where ATVs are sold (dealers) and recreationally used (tracks). Thus, this study was an exploratory study on the safety promotion and recommendations by ATV dealers and track owners in Kansas.

## Methods

### Study Design and Study Population

This was a cross-sectional telephone survey of ATV dealers and track owners in the state of Kansas. A list of ATV dealers and track owners was compiled from a Google™ search of ATV dealers and tracks in Kansas. The survey consisted of predominantly yes or no questions regarding the respondents’ safety promotion practices and included promotion and sale of various safety equipment (questions were specific to safety item, such as Department of Transportation (DOT) or Snell certified helmet) and provision of ATV safety information. Respondent characteristics such as ATV use and experience were included. Dealer respondents were queried regarding their experience with ATV accidents. The identified survey participants were contacted once and the dealer or track owner, or someone who was knowledgeable about the operation was requested to respond to the survey. The informed consent process was conducted verbally and completion of the survey indicated consent. The project was approved by the Wichita State University Institutional Review Board.

### Data Analysis

Data were reported descriptively using frequencies (percentages). Significance tests were conducted with the chi-square test of association and Fisher’s exact statistics. The data were analyzed with SPSS for Windows, Version 23.0.

## Results

### Survey Respondents

Thirteen of forty-one dealers participated in the survey for a response rate of 32% (Table 1). Half of respondents (7/13) reported being an ATV salesperson. Most dealers (10/13) sold ATVs as secondary products (such as car dealership) with ATV sales ranging from five to 200 annually. Few dealer respondents (2/13) reported owning an ATV; most (11/13) reported personally riding ATVs. Most respondents (11/13) reported knowing someone involved in an ATV accident.

Five of sixteen ATV tracks participated in the survey for a response rate of 31%. Most respondents (3/5) reported being a track owner/manager. Only one of the track respondents reported owning an ATV; while three reported personally riding ATVs. Most respondents (3/5) reported knowing someone involved in an ATV accident.

### ATV Dealer Safety Promotion

All ATV dealer respondents reported asking a buyer how ATVs will be utilized, but less (11/13) asked the age of the primary rider and fewer (5/13) asked about secondary riders (Table 2). Only half of dealer respondents (7/13) reported it was their professional responsibility to provide ATV safety education to buyers. Dealer belief regarding professional responsibility to educate on safety was associated with other characteristics or safety promotion significantly.

### ATV Track Safety Promotion

All ATV track respondents (100%) reported requiring riders to wear helmets (Table 3). Over half (3/5) reported specific helmet requirements. Of those, all required Department of Transportation (DOT) certified and most (2/3) required Snell certified helmets. No respondents reported requiring over the ankle boots or chest protectors, but two require goggles and closed toe shoes. Two of the 5 track respondents also reported providing safety courses and more than half (3/5) offered additional safety information. Most track respondents (4/5) agreed it is their professional responsibility to educate riders on ATV safety; the only track respondent who did not agree did not own/ride ATVs nor knew anyone involved/killed in an ATV-related accident.

## Discussion

The aim of this study was to describe ATV safety promotion (as sales or use of safety gear or provision of education) at the point of sale or track use in the state of Kansas. While all dealer respondents reported recommending safety gear and selling head protection, not all sell other safety gear such as body protection. Further, safety courses and skills tests are not commonly reported safety promotion practices at point of sale. Self-reported safety practices by participating tracks include all requiring head protection, less have requirements regarding age of rider and size of rider to ATV. Few ATV dealer or track respondents report providing safety information or courses.

Historical studies such as Percy and Duffy^18^ reporting ATV related injuries and Warda and colleagues^1^ reporting safety behaviors have called for preventive and safety measures such as consistent use of safety gear, mandatory rider training, as well as consumer and dealer education. Congruently, recent literature also concluded that safety precautions can reduce injury related costs^5^ and recommended preventative guidelines^6^ or initiatives^10^ to reduce ATV related injuries. ATV dealers and track owners may be open to displaying ATV safety information similarly to agribusinesses.^17^ Public health campaigns through influential change agents, such as ATV dealers and track owners, may serve to increase awareness of protective safety practices, such as consistent use of helmets.^4^,^7^ Healthcare providers who treat patients using all-terrain vehicles should be aware of the scarcity of safety promotion and also consider rider safety education.

### Study Limitations

The results of this descriptive study may be limited by selection bias (Google™ search compiled list), response bias (ATV dealers and track owners in Kansas, predominantly rural state), and variability among dealers who sell ATVs and may not generalize to other dealers and track practices. Further, generalizability is limited by low response rates from both dealers (32%) and tracks (31%), however, this is the first research to assess safety promotion practices at the dealer and track level.

### Future Research

Future research should delve deeper into safety promotion practices at the state and national levels. The evaluation of ATV safety programs would be valuable to determine the types of programs that are successful in preventing ATV related injuries and mortality. A quality analysis of ATV safety materials (manufacturer, house-developed, and public health promotion) may be of value to determine consumer usability.

## Conclusion

All-terrain vehicle dealers are recommended to promote ATV safety, but typically such promotion is limited to the sales of safety gear (helmets and gloves) or the provision of manufacturer provided safety materials. Further, ATV dealers report rarely offering skills tests or safety courses to buyers. Regarding ATV tracks, helmet protection is standard, however, ATV riding practices (age of rider, size of ATV) usually are not monitored. Moreover, only about half offered safety courses or materials. In Kansas, safety promotion at the point of sale or track level could be improved to increase public awareness of ATV safety practices.

## Figures and Tables

**Table 1:** Characteristics of survey respondents. Data are reported in frequencies (percentages).

	Dealers (N=13)	Tracks (N=5)

Personally ride ATV	11 (85)	3 (60)

Personally own ATV	2 (15)	1 (20)

Personally involved in ATV accident	8 (62)	1 (20)

Know someone in accident	11 (85)	3 (60)

Know someone disabled in an accident	4 (31)	NA

Know someone killed	3 (23)	NA

Agrees		
	
States laws should be stricter	3 (23)	NA
	
Professional responsibility to educate on safety	7 (53)	4 (80)

* NA = Question not asked in survey.

**Table 2:** Dealer respondents’ self-reported safety promotion (N=13).

*Safety Gear*	N (%)
Recommend	13 (100)
Sell	13 (100)
Sell Head Protection	
DOT or Snell Certified	13 (100)
Open Face with Shield	12 (92)
Open Face without Shield	12 (92)
Motocross Style	13 (100)
Sell Body Protection	
Ankle Boots	10 (77)
Chest Protectors	9 (69)
Gloves	13 (100)
Clothing	12 (92)
***Safety Information at Purchase***	
Inquire age of rider	11 (85)
Offer courses	4 (31)
Offer safety information	4 (31)
Perform skills test	2 (15)

**Table 3:** Track respondents’ self-reported safety promotion (N = 5).*

*Personal protection*	
Require head protection	5 (100)
DOT or Snell Certified	3 (60)
Require body protection	
Ankle Boots	0 (0)
Chest Protectors	0 (0)
Goggles	2 (40)
Closed toe shoes	2 (40)
*Provide safety education*	
Safety courses	2 (40)
Safety information	3 (60)
*Enforce track safety rules*	
Age limits	2 (40)
Allow multiple riders	2 (40)
Monitor size of rider to ATV	1 (20)
Limitations on engine cc	3 (60)
Provide medical personnel during races	3 (60)

* Frequency (percentage) reported.

DOT = Department of Transportation.
